# Genomic resources for the endangered Hawaiian honeycreepers

**DOI:** 10.1186/1471-2164-15-1098

**Published:** 2014-12-12

**Authors:** Taylor Callicrate, Rebecca Dikow, James W Thomas, James C Mullikin, Erich D Jarvis, Robert C Fleischer, NISC Comparative Sequencing Program

**Affiliations:** Center for Conservation and Evolutionary Genetics, Smithsonian Conservation Biology Institute, Washington, DC 20008 USA; Department of Animal and Avian Sciences, University of Maryland, College Park, MD 20742 USA; National Human Genome Research Institute, NIH, Bethesda, MD 20892 USA; Department of Neurobiology, Howard Hughes Medical Institute, Duke University Medical Center, Durham, NC 27710 USA

**Keywords:** Genome, Hawaiian honeycreepers, SNP, RAD tags, Drepanidines, *Hemignathus virens*

## Abstract

**Background:**

The Hawaiian honeycreepers are an avian adaptive radiation containing many endangered and extinct species. They display a dramatic range of phenotypic variation and are a model system for studies of evolution, conservation, disease dynamics and population genetics. Development of a genome-scale resources for this group would augment the quality of research focusing on Hawaiian honeycreepers and facilitate comparative avian genomic research.

**Results:**

We assembled the genome sequence of a Hawaii amakihi (*Hemignathus virens*),and identified ~3.9 million single nucleotide polymorphisms (SNPs) in the genome. Using the amakihi genome as a reference, we also identified ~156,000 SNPs in RAD tag (restriction site associated DNA) sequencing of five honeycreeper species (palila [*Loxioides bailleui*], Nihoa finch [*Telespiza ultima*], iiwi [*Vestiaria coccinea*], apapane [*Himatione sanguinea*], and amakihi). SNPs are distributed throughout the amakihi genome, and the individual sequenced shows several large regions of low heterozygosity on chromosomes 1, 5, 6, 8 and 11. SNPs from RAD tag sequencing were also found throughout the genome but were found to be more densely located on microchromosomes, apparently a result of differential distribution of the particular site recognized by restriction enzyme *Bse*XI.

**Conclusions:**

The amakihi genome sequence will be useful for comparative avian genomics research and provides a significant resource for studies in such areas as disease ecology, evolution, and conservation genetics. The genome sequences will enable mapping of transcriptome data for honeycreepers and comparison of gene sequences between avian taxa. Researchers will be able to use the large number of SNP markers to genotype honeycreepers in regions of interest or across the whole genome. There are enough markers to enable use of methods such as genome-wide association studies (GWAS) that will allow researchers to make connections between phenotypic diversity of honeycreepers and specific genetic variants. Genome-wide markers will also help resolve phylogenetic and population genetic questions in honeycreepers.

**Electronic supplementary material:**

The online version of this article (doi:10.1186/1471-2164-15-1098) contains supplementary material, which is available to authorized users.

## Background

Avian genome sequences were first obtained for well-studied model systems for which there was a long history of multidisciplinary research, namely the chicken *Gallus gallus*[1] and zebra finch *Taeniopygia guttata*[2]. But now genomes are starting to appear along lines of interest such as other agricultural species (turkey, *Meleagris gallopavo*[3]), members of adaptive radiations (Darwin’s medium ground finch, *Geospiza magnirostris*[4]), species with traits of interest such as vocal learning (budgerigar, *Melopsittacus undulatus*[5]) and systems with possible incipient speciation (*Ficedula* flycatchers [6]). Genome-scale resources for non-traditional model organisms have become a reality over a short period of time, due in a large part to the commercialization of sequencing-by-synthesis (also called next-generation sequencing) technology [7]. Initial examinations of these genomes have revealed that there is a high degree of synteny among avian species, confirming hypotheses from cytogenetic studies [8]. Although 40 million years of evolution separate chickens and turkeys, only 30 minor chromosome rearrangements were detected between the two and their karyotypes are strikingly similar [3]. Chicken and zebra finch (perhaps 100 million years diverged [9]) also exhibit a high degree of synteny and conservation of karyotype [2]. However, recent work shows that small inversions may be common when comparing distantly-related avian taxa [10].

There are over 5,000 passerine species with many unique traits and adaptations [11]. Each additional passerine genome [2, 4, 6] that is sequenced offers an opportunity to identify different genes under selection and to elucidate the mechanisms underlying avian adaptations [4]. The Hawaiian honeycreepers are an endemic Hawaiian passerine adaptive radiation in the Cardueline finch subfamily Drepanidinae [12], and display a tremendous diversity of plumages, beak shapes (some unique to this radiation) and niches [13]. Molecular analyses indicate that the radiation is sister to the Eurasian *Carpodacus* rosefinches, and dates to about 5.7 million years ago [12, 14]. Adaptive radiations have long been recognized for their value as evolutionary case studies and their usefulness in understanding adaptive evolutionary processes. The Hawaiian honeycreepers have the special characteristic that the history of their radiation is integrated with the geological history of the Hawaiian Islands. Patterns in honeycreeper divergence appear to be linked to the pattern of island emergence [12], which has been well-documented as part of a volcanic time series [15]. Because this unusual geology provides a well-defined timeline, honeycreepers are a good system for estimation of rates of molecular evolution [14].

Unfortunately, of the 33 described historical honeycreeper species (plus over 17 species known only from subfossil material) [13], roughly two-thirds are now extinct, largely from human-related impacts such as habitat loss, introduced mammalian predators and vectored pathogens [16]. Study of the evolution of disease resistance is an area that will especially benefit from genome-wide markers. In particular, honeycreepers appear extremely susceptible to introduced diseases such as avian malaria (*Plasmodium relictum*) and avian poxvirus, both vectored by an introduced *Culex* mosquito [17–19]. Most extant honeycreepers are limited to higher elevations free from mosquitoes and disease [20]. However, a few species, most notably the Hawaii amakihi (*Hemignathus virens*), can survive with chronic malaria infection, exhibiting tolerance or resistance to the disease [21–23]. A few studies suggest that strong selective pressure from malaria resulted in rapid evolution of disease tolerance in certain low-elevation Hawaii amakihi populations and that resistance may be spreading amongst low-elevation amakihi, although it is unknown whether resistance arose once or simultaneously in multiple source populations [24]. Understanding the source and mechanism of disease resistance in amakihi is a priority research area using the SNP markers. Such work is needed to improve our strategies for identifying and preserving the most viable populations of many species threatened by invasive pathogens.

Our objective in this study is to characterize the genome of a Hawaiian honeycreeper, the Hawaii amakihi (*Hemignathus virens*), and to develop and assess a set of genome-wide SNP markers to enable both phylogenetics-scale and fine-scale investigations about adaptive evolution and population genetics. We used two sequencing-by-synthesis approaches and then performed a hybrid assembly to create a draft Hawaii amakihi genome sequence. The Hawaii amakihi, in addition to being a member of the honeycreeper adaptive radiation, serves as an ecological model for disease transmission due to its variable responses to infection by avian malaria [21, 22]. The individual selected for the genome sequence had a high level of infection, but had been recaptured several times, indicating persistence despite a chronic, intense malaria infection. To increase the utility of markers for broader topics of study, we combined de-novo genome sequencing with a reduced representation sequencing method (restriction site-associated DNA, or RAD) to identify and map SNP polymorphisms isolated from four additional honeycreeper species. In addition to facilitating research into honeycreeper evolution and disease resistance, the draft amakihi genome will contribute to knowledge of avian genome biology and improve the pool of resources for comparative genomic study.

## Results and discussion

### Genome assembly

Our hybrid approach utilized both Roche/454 and Illumina technology (see Table 1). Illumina sequencing of the amakihi genome generated approximately 31 GB of data composed of over 300 million read pairs (174.24 × 10^6^ 2 × 101 bp, 4.08 × 10^6^ 2 × 151 bp and 152.67 × 10^6^ 101 × 88 bp pass-filter reads) and represented an approximately 60-fold coverage of the genome. The 454 data comprised 2 – 3x coverage, with 458 bp average read length. This is a substantially larger dataset than for the first avian genome, chicken, which was done using 11 million Sanger reads with 6.6- fold coverage [1].

**Table 1:** **Summary of input for genome assembly**

Platform	Read type	Reads/read pairs
Illumina	2 × 151	3.93 × 10^6^
Illumina	2 × 101	86.97 × 10^6^
454	Fragment	3.64 × 10^6^

The hybrid assembly used the full 2x 454 coverage and ~19x Illumina coverage (see Table 1), similar to the process for turkey which used ~5x 454 and ~25x Illumina GAII [3]. We used only a portion of the total Illumina data to avoid overwhelming the information from the 454 reads; limiting the data volume was also necessary to stay within the memory limits of the computer used (512 GB RAM). Contigs were ordered and oriented and extended into scaffolds by aligning to the zebra finch genome sequence. In this way, amakihi genotypes at each zebra finch genomic position were determined. Genotype calls were generated using only high-quality (Phred-like Q20 or above) bases in the mapped reads. An MPG [25] score cutoff of ≥ 10 is expected to yield high-quality genotypes with >99.84% concordance with those from an Illumina Infinium genotyping assay [26].

The structure of avian genomes in general appears to be relatively undisturbed with regard to rearrangements, resulting in high degree of synteny among a variety of bird species [27]. This property has been observed when comparing turkey [3] and *Ficedula* flycatcher to chicken [28]. Our use of zebra finch as a template for aligning and assembling the amakihi genome is justified, in part, by the relatively recent divergence (33.5 million years) of the species [29]. In fact, the *Ficedula albicollis* genome shows remarkably strong synteny with chicken despite perhaps 100 million years of evolutionary distance [28]. However, on a more localized scale, Ficedula flycatchers show many small rearrangements with respect to zebra finch [10]. If similar rearrangements have occurred between zebra finch and amakihi, then our assembly could be different from the true amakihi genome sequence.

The N50 value of contigs from the hybrid assembly was 23 kb, and 50 kb for scaffolds. This value is smaller than for other recently published bird genomes; for example, Darwin’s finch had a 382 kb scaffold N50 [4], and the value for flycatcher was 7.3 Mb [6]. Additional sequencing libraries of larger insert sizes would perhaps have resulted in larger N50 values; however, this was effectively accomplished by ordering the contigs relative to the zebra finch genome. Total assembly size of the amakihi genome was approximately 1 Gb, similar in size to other bird genome assemblies (for example, 1.05 Gb for chicken [1], 1.2 Gb for zebra finch [2], 1.1 Gb for turkey [3], 1.1 Gb for collared flycatcher [6], and 991 Mb (true size estimated to be 1.25 Gb) for Darwin’s medium ground finch [4]). We believe that our amakihi genome is relatively complete because the assembly size is similar to other bird genomes. We further tested this assumption by aligning zebra finch sequences to selected portions of the honeycreeper assembly and determining the percentage that successfully aligned. Overall for the numbered chromosomes (not including random, chrM or chrUn), 86.33% of zebra finch sites could be aligned (mean: 77.26 ± 17.69; see Table 2).From this alignment we also calculated the genetic distance between amakihi and zebra finch as 0.0905 (Kimura two parameter model; see Table 2). It is possible that this value is underestimated since regions greatly diverged between amakihi and zebra finch may not have successfully mapped to the zebra finch reference.

**Table 2:** **Alignment statistics for zebra finch and amakihi against amakihi genome**

Chrom.	% of zebra finch sites aligned (non-N)	% of amakihi sites aligned (non-N)	Uncorrected p-distance	Kimura two parameter model distance
chr1	87.54	90.67	0.09	0.09
chr10	87.66	91.68	0.08	0.08
chr11	87.65	89.89	0.08	0.08
chr12	85.83	90.77	0.08	0.08
chr13	84.46	89.64	0.08	0.09
chr14	88.44	89.85	0.08	0.08
chr15	83.49	87.47	0.08	0.08
chr17	83.60	87.63	0.08	0.08
chr18	81.18	79.45	0.08	0.09
chr19	81.35	86.12	0.08	0.08
chr2	87.90	90.72	0.09	0.09
chr20	81.75	87.00	0.08	0.08
chr21	77.05	81.73	0.08	0.09
chr22	63.61	70.26	0.10	0.11
chr23	71.64	78.85	0.09	0.09
chr24	72.44	78.98	0.09	0.10
chr25	72.01	75.09	0.10	0.11
chr26	77.91	79.94	0.09	0.09
chr27	65.53	73.24	0.09	0.10
chr28	75.92	72.64	0.09	0.09
chr3	91.56	92.23	0.08	0.09
chr4	87.78	91.91	0.09	0.09
chr5	87.80	91.76	0.08	0.09
chr6	85.38	91.11	0.08	0.09
chr7	83.72	91.19	0.08	0.09
chr8	87.09	92.25	0.08	0.08
chr9	83.21	90.02	0.08	0.09
chr1A	88.13	90.70	0.09	0.09
chr1B	68.96	78.58	0.10	0.11
chr4A	84.31	90.13	0.08	0.09
chrLG2	19.65	64.27	0.15	0.17
chrLG5	5.49	62.13	0.15	0.17
chrLGE22	73.78	79.21	0.09	0.10
chrZ	82.55	84.25	0.10	0.11

**Figure 1:**
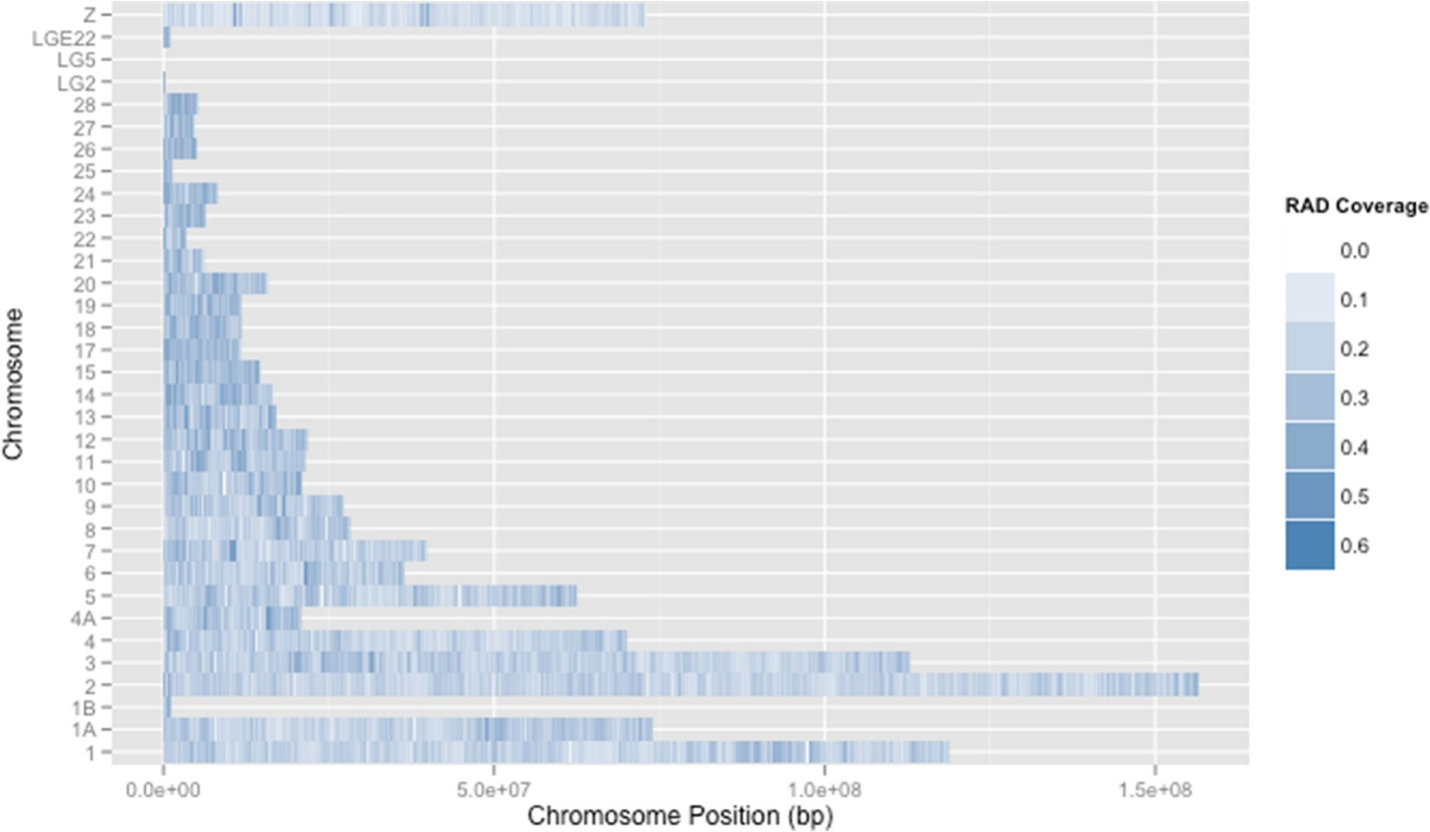
**RAD coverage of amakihi chromosomes.** Colors indicate proportion of 100 Kb bins covered by at least 1 bp of RAD sequence.

A total of 1.04 Gb of the amakihi assembly was localized to 34 chromosomes by aligning contigs and scaffolds to zebra finch chromosomal sequences. Although previously assembled avian genomes have taken advantage of linkage maps from the same species for chromosome assignment (i.e., 93% assigned to chromosomes for turkey [3]), alignment to other genomes has also been used. For *Ficedula albicollis*, 73% of the genome sequence was assigned to chromosomes using the flycatcher linkage map; by comparing conserved organization with zebra finch, a total of 89% could be assigned [6]. As was the case for turkey [3] and chicken [1], most of the honeycreeper chromosomes are microchromosomes that cannot always be distinguished by size alone (see Figure 1, which shows relative chromosome lengths). The draft amakihi genome sequence is available in FASTA format in the NCBI repository, BioProject: PRJNA252695

After assembly, a larger number of Illumina reads were aligned back to the assembled genome to a depth of ~47.6x for the autosomes and ~25x for the Z chromosome to identify and call SNPs. Nucleotide diversity (π) on the autosomes ranged from 0.0022 on chromosome LGE22_random to 0.0113 on chromosome LG5 (Table 3: summary of nucleotide diversity by chromosome).

**Table 3:** **Nucleotide diversity by chromosome**

Chromosome	Homozygous sites	Heterozygous sites	π
chr1	112,544,959	485,712	0.0043
chr10	19,502,766	67,127	0.0034
chr10_random	181,773	748	0.0041
chr11	20,339,491	68,262	0.0033
chr11_random	205,478	795	0.0039
chr12	19,966,665	77,365	0.0039
chr12_random	142,337	627	0.0044
chr13	15,608,448	63,230	0.0040
chr13_random	2,273,196	6,948	0.0030
chr14	15,783,392	62,881	0.0040
chr14_random	119,916	586	0.0049
chr15	13,395,570	47,525	0.0035
chr15_random	336,675	1,356	0.0040
chr16_random	28,278	132	0.0046
chr17	10,789,469	43,477	0.0040
chr17_random	69,369	290	0.0042
chr18	11,093,387	30,844	0.0028
chr18_random	393,813	1,922	0.0049
chr19	10,638,978	41,233	0.0039
chr19_random	61,004	162	0.0026
chr1A	70,419,613	301,663	0.0043
chr1A_random	429,913	1,856	0.0043
chr1B	900,172	4,219	0.0047
chr1B_random	100,455	761	0.0075
chr1_random	150,801	806	0.0053
chr2	149,097,369	652,060	0.0044
chr20	14,291,352	57,186	0.0040
chr20_random	138,194	682	0.0049
chr21	5,425,030	24,579	0.0045
chr21_random	1,777,800	4,856	0.0027
chr22	2,908,707	11,322	0.0039
chr22_random	657,788	3,832	0.0058
chr23	5,370,519	23,530	0.0044
chr23_random	370,728	2,169	0.0058
chr24	7,044,699	31,458	0.0044
chr24_random	74,717	253	0.0034
chr25	1,142,233	4,993	0.0044
chr25_random	345,747	2,115	0.0061
chr26	4,582,739	19,099	0.0042
chr26_random	1,375,049	7,314	0.0053
chr27	3,929,203	14,589	0.0037
chr27_random	187,008	875	0.0047
chr28	4,923,374	18,553	0.0038
chr28_random	158,967	1,285	0.0080
chr2_random	408,633	1,750	0.0043
chr3	110,159,365	497,976	0.0045
chr3_random	850,964	4,677	0.0055
chr4	65,570,862	294,828	0.0045
chr4A	18,959,367	64,240	0.0034
chr4A_random	68,624	262	0.0038
chr4_random	4,413,118	21,779	0.0049
chr5	58,574,618	240,015	0.0041
chr5_random	1,912,995	11,030	0.0057
chr6	33,425,145	73,863	0.0022
chr6_random	1,513,279	8,220	0.0054
chr7	35,848,910	146,136	0.0041
chr7_random	205,374	1,023	0.0050
chr8	25,953,825	94,369	0.0036
chr8_random	4,504,789	14,345	0.0032
chr9	24,645,966	100,108	0.0040
chr9_random	121,289	669	0.0055
chrLG2	27,825	241	0.0086
chrLG5	1,309	15	0.0113
chrLGE22	781,559	2,626	0.0033
chrLGE22_random	75,448	170	0.0022
chrUn	7,431,999	41,937	0.0056
chrZ	68,235,778	9,906	0.0001
chrZ_random	2,178,358	2,162	0.0010
chrUn2	21,894,126	153,065	0.0069

Because in birds females are the heterogametic sex (we sequenced a female) chromosome Z should in theory have no heterozygous sites except in pseudo autosomal regions. Our data show about 0.017% of the total sequence sites assigned to Z and Z random are heterozygous (9,906 heterozygous sites on Z and 2,162 on Z random) versus 0.417% for sites on autosomal chromosomes. These false positives on the Z could be attributed to mismapping of paralagous reads or misassignment of autosomal segments to the Z and Z random chromosomes. The false positive rate on Z/Z random is an approximate indicator of the false positive rate elsewhere in the genome because mismapping of paralagous sites could have occurred for autosomal chromosomes as well.

**Figure 2:**
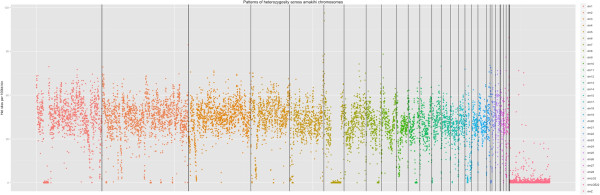
**Patterns of heterozygosity across amakihi chromosomes.** Each dot represents the count of heterozygous sites in a 100 kb bin. Colors represent different chromosomes, which are also separated by vertical lines. Note stretches of low heterozygosity on chromosomes 1, 5, 6, 8, and 11.

Approximately 3.9 million SNP sites were discovered in the assembled amakihi genome, or approximately one SNP every 256 bp. This is similar to results for the flycatcher, where 3.66 million SNPs (one per 330 bp) were identified in one individual [6]. Heterozygosity was characterized for each chromosome by counting the number of heterozygous sites in 100 kb bins along each chromosome (Figure 2). Large stretches of extremely low variability (nearly zero heterozygosity) were observed on five chromosomes (1, 5, 6, 8 and 11). Coverage for these regions was not different than for other sites in the genome. They ranged in size from 2 Mb on chromosome 5 to 17.9 Mb on chromosome 6 and together made up 3.51% of the genome sequence (Figure 2). Large stretches of low heterozygosity were also observed on turkey chromosomes 1 and 3 and were interpreted as IBD (identical by descent; having come from a recent common ancestor) haplotypes [30]. The turkeys described in that study were from domestic lines that had been subjected to many generations of artificial selection, so finding IBD regions was not unexpected. In the case of the amakihi, which has a relatively large population size, inbreeding is not expected. For inbreeding between first order relatives (i.e., parent-child) approximately 25% of the genome would be expected to show large homozygous stretches, while inbreeding of second order relatives (such as uncle-niece/aunt-nephew) would result in about 12.5%. To differentiate between the effects of inbreeding and selection, we would need to determine the probability of SNP loci in the low heterozygosity regions being IBD or identical by state (IBS; sharing the allele by chance rather than inheriting it from the same ancestor). As we obtain more data from other amakihi, we will be able to calculate allele frequencies for the loci in question and be able to calculate IBD/IBS probability for the low heterozygosity regions. These regions could possibly represent signatures of selective sweeps in the evolutionary history of the amakihi, or be the result of inbreeding, although the latter may be less likely given the relatively high variation found in amakihi from the same locality as 1771-10606, the individual whose genome is presented here. We compared gene classifications within each homozygous region to those on the rest of each respective chromosome using Ensembl annotations for the zebra finch (http://www.ensembl.org/Taeniopygia_guttata/Info/Index). No substantial difference was observed.

### RAD data

The RAD tag method involves digesting genomic DNA with a restriction enzyme and sequencing fragments (tags) of DNA adjacent to restriction sites [31]. We sequenced RAD tags for six individuals of four honeycreeper species in addition to the same amakihi for which we obtained the genome. This method yielded a wide range of sequences per individual, with an average of 7,596,336 post quality filtering (range: 319,559 – 24,263,032; see Additional File 1). We attribute the large range of number of reads to stochastic factors and variable sample DNA quality, as all other parameters (DNA quantity, library preparation protocol, pooled for sequencing in equimolar ratios) were the same between samples. RAD sequences were analyzed following two protocols: without a reference genome, using the Stacks pipeline, or utilizing the amakihi sequence as a reference for assembly and genotype calling. Raw reads for each individual in FASTQ format have been uploaded to NCBI (BioProject 252695) and will be available after publication of this article.

By using Stacks to assemble and genotype RAD sequences, we found 309,957 loci with 173,553 passing our filters, 17,513 of which were variable loci containing at least one SNP site within or between individuals (see Table 4). There were, on average, 40,270 loci per species passing our filters (range: 2,351 – 123,623) and 3,996 SNPs per species (range: 515 – 12,422); i.e., about 10% of loci contained SNP(s). Only 473 stacks with 109 total SNPs were shared by at least three of the honeycreeper species.

**Table 4:** **Stacks results after quality filtering**

Species	Number of stacks loci	Number of SNPs	Number of variable loci
Apapane	17,357	680	573
Nihoa Finch	3,004	841	577
Palila	2,351	515	354
Iiwi	55,014	5,523	4,197
Amakihi	123,623	12,422	9,536

Since we had both RAD and genome data for the same individual amakihi, we compared genotype calls from Stacks to known values from the genome sequence. With a minimum stack depth requirement of nine, only 0.8% of Stacks SNP calls differed from the genome value.

### RADs with a reference

**Figure 3:**
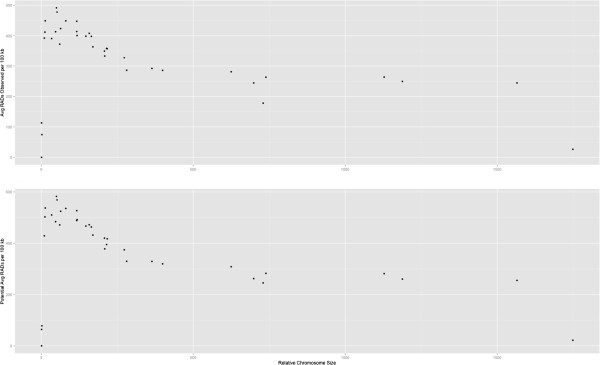
**Relationship between relative chromosome size and RAD density.** Top panel shows the density of RADs based on our RAD sequencing (see Figure 1); bottom panel shows the density of restriction sites and potential RADs based on in silico digest of the amakihi genome.

We also analyzed RAD data with the benefit of the amakihi reference sequence. Restriction cut sites, and therefore RAD sequences, are expected to be randomly, not evenly, distributed across the genome [32]. When aligning honeycreeper RAD sequences to the amakihi genome, we observed a denser distribution of RADs on the microchromosomes (Figures 1 and 3). We found the same pattern of non-random distribution of restriction sites based on an *in silico* restriction digest of the amakihi genome (Figure 3). One possible explanation for this is that the microchromosomes of avian species are commonly more gene-dense than the macrochromosomes, with a higher GC content [33–35], and restriction enzymes tend to have a high proportion of GC content in their binding site [36]. The enzyme used in this study, BseXI, contains 80% GC in its 5 bp recognition site, making this a plausible explanation. Alternatively, there may be more repetitive DNA sequences in macrochromosomes, and the repetitive sequences might not contain the BseXI recognition site. Being able to align RADs to a reference provides an advantage for researchers who may wish to select a smaller number of RAD SNP sites for genotyping, as the spacing and location of specific markers makes it easier to narrow down to only the necessary ones.

We used the Burrows-Wheeler Aligner (BWA, [37]) and the Genome Analysis Toolkit (GATK, [38]) in conjunction with the amakihi reference sequence to identify inter- and intraspecific SNPs using the RAD sequences. Using this method, we identified 172,085 SNP sites with 156,486 passing quality filters (See Table 5). After filtering, there were, on average, 52,348 sites with a known genotype identified per sample (range: 15,800 – 110,844) including an average of 1,727 heterozygous sites per sample (range: 291 – 4,137). 9,714 non-reference sites were shared by at least four samples.

**Table 5:** **SNP sites discovered by comparison to the honeycreeper reference. Filtered for qual > 30 and depth >6**

Sample	Positions with known genotype	Heterozygous sites	Sites with non-reference allele	Private non-reference alleles
Nihoa_Finch_1	93,646	2,864	91,038	15,715
Nihoa_Finch_2	110,844	4,137	108,297	30,168
Iiwi	15,800	291	12,685	524
Palila_1	17,511	571	14,580	841
Amakihi	50,489	2,202	22029	3,587
Palila_2	25,795	299	22,529	1,664

Compared to analyzing without a reference, the BWA-GATK pipeline resulted in more SNPs identified for Nihoa finch, fewer for iiwi, about the same for palila, and fewer for amakihi.

### Interspecies comparisons

**Figure 4:**
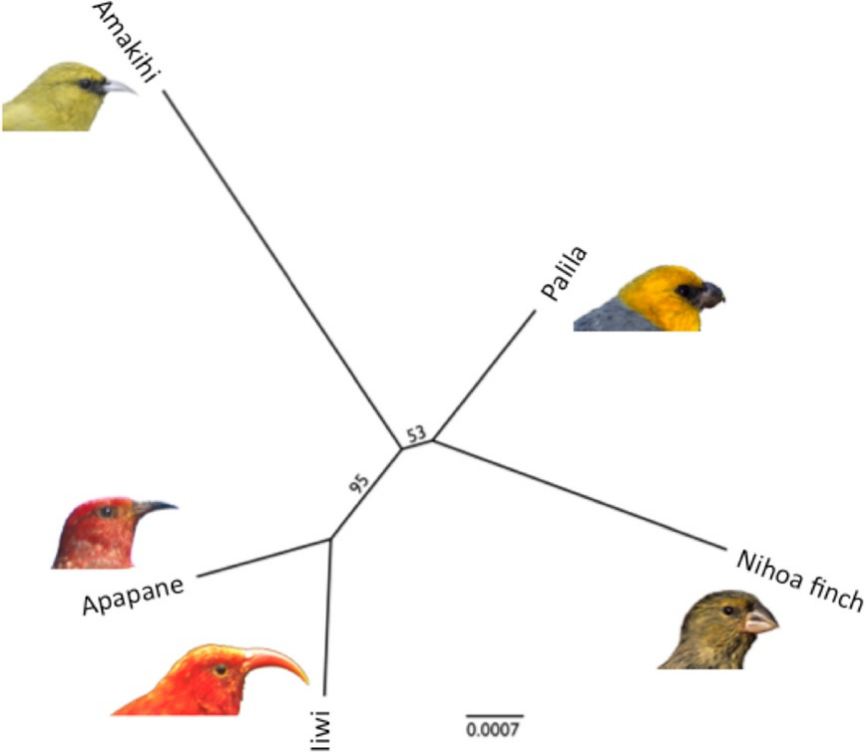
**Reconstructed maximum likelihood tree of relationships of the five study species based on RAD sequences.**

We performed a phylogenetic analysis to demonstrate the utility of RAD sequences for determining relationships amongst taxa. PyRAD [39] was used to identify and homologize RAD sequences with 10X or higher coverage present in three or more taxa, which produced 38,889 bp. A maximum likelihood analysis was performed on these data in Garli [40] with 1,000 bootstrap replicates and the relationships of the five species are shown (Figure 4). This analysis recovered the expected topology with good support for the iiwi/apapane relationship. Support for the palila/Nihoa finch node was low, perhaps as a result of the deeper divergence between these species than between iiwi and apapane, and the shorter internode between this clade and the amakihi clade [12].

### Applications of honeycreeper genomic resources

Herein, we describe a draft genome sequence for the Hawaii amakihi and associated genomic resources for Hawaiian honeycreepers including approximately 3.9 million SNPs within the amakihi genome and over 150,000 SNPs within and between amakihi and four other honeycreeper species. Honeycreepers are an important model system for many questions in evolutionary biology, and the SNP markers will facilitate a wide range of future studies in ongoing and new research areas. Being genome-enabled both enhances the resolution of current research methods (for example, fully resolving the honeycreeper phylogeny) and also opens up new analyses that weren’t possible before (such as GWAS for malaria tolerance). Some of the important questions which may be addressed include: how do rates of sequence evolution vary among different classes of DNA; what genes or genome regions are involved in speciation, adaptation or evolution of tolerance or resistance to disease; and how much adaptive potential exists in a population after demographic decline or fragmentation?

Studies of the evolutionary relationships of honeycreepers (for example [41–43]) have been limited by available technology and methods, as well as by rapid speciation and low levels of sequence divergence. Early molecular studies used allozyme electrophoresis [14, 44], restriction fragment length polymorphism of mitochondrial DNA [45], and relatively short DNA sequences [14, 46, 47] to only marginally resolve nucleotide substitution rates and relationships within the honeycreepers. Larger molecular datasets, such as one with entire mitochondrial genomes and 13 nuclear loci (>15 Kb) more adequately resolved the phylogeny, and estimated rates of sequence evolution and a split from a cardueline finch lineage at 5.7 Mya [12]. Re-evaluating the honeycreeper phylogeny with a larger, more comprehensive dataset will allow researchers to investigate the pattern and tempo of evolution in this radiation. With genome-wide markers, it will be possible to connect genomic regions with specific adaptive traits across the phylogeny. Because precise geological information about the Hawaiian Islands provides a framework for dating evolutionary events, the honeycreeper radiation can provide unique insights into the evolutionary process. What is learned from honeycreepers can also be compared with other avian adaptive radiations such as Darwin’s finches [4] to further our understanding of the evolutionary process overall.

The ability to use analytical tools that connect genotypes to traits, such as GWAS [48, 49]) is a key benefit of the honeycreeper genomic marker set. These methods require large numbers of markers and were previously only useful for genome-enabled model organisms. Such techniques may allow identification of genes or regions implicated in disease resistance or specific adaptive traits; when such information is combined with results in other taxa, it contributes to our overall understanding of molecular mechanisms. This is also a first step towards investigating what happens to the genetic diversity in adaptively important genes or regions when species decline and become endangered. Identifying key genomic regions for disease resistance or adaptation could help focus conservation efforts towards preserving genetic variation in those areas and provide guidance for genetically-based population management decisions.

Hawaiian honeycreepers are also a model to investigate the response of genetic variation to human caused population decline, fragmentation and founder effects. For example, the Hawaii akepa (*Loxops coccineus coccineus*) occupies < 10% of its historical range in fragmented habitat and is a magnitude less populous than before its decline, yet contemporary samples show the same level of mitochondrial genetic diversity as in specimens sampled > 100 years ago and no significant differentiation between fragmented populations is detected [46]. In another case, several founder populations of Laysan finch (*Telespiza cantans*) have been established on Pearl & Hermes reef and microsatellite data reveal that these have become genetically differentiated from the Laysan population and, to some extent, from each other [50]. Finally, Hawaii amakihi, which have a relatively large population size, exhibit a rather unique elevational structuring, with populations from high elevation genetically differentiated from those at low elevation; data from museum skins suggest that this was also true historically. This elevational pattern is not found in contemporary iiwi (*Vestiaria coccinea*) or apapane (*Himatione sanguinea*) populations [24]. Using the more comprehensive SNP marker set will provide the power to start looking at selection and adaptation to anthropogenic caused change in these species.

## Conclusions

Our results provide a set of genomic resources for Hawaiian honeycreepers that will facilitate research on disease interactions, metapopulation dynamics, adaptive radiations, and genome evolution. The amakihi genome sequence will enable comparative studies of avian genomes and is an important contribution as it represents one of the more than 5,000 passeriform species, a group for which there are currently only three other genomes available in the literature [2, 4, 6]. The results yield a large number of genome wide markers, both from heterozygous sites in the sequenced individual and discovered using RAD tags with other honeycreeper species. We have demonstrated their potential phylogenetic utility based on a tree of relationships between honeycreeper species used in our RAD analysis that matches expectation based on previous molecular phylogenetic analyses [12]. Heterozygosity measures for the individual sequenced, a malaria-resistant amakihi, indicate some regions of potential selective sweeps that could be of interest for study of malaria resistance. These regions are being targeted for resequencing in populations of malaria resistant and susceptible amakihi. The markers could also be used to identify regions of divergence among honeycreeper species to help elucidate the speciation process [6].

## Methods

### Study samples

A single female amakihi (*Hemignathus virens*) was sequenced for genome assembly (USGS aluminum band 1771-10606, sampled 22 February 2002 at Nanawale, Hawaii Island). Although it has been typically preferred to use an inbred individual for genome sequencing to simplify assembly, the possibility of high-coverage sequencing-by-synthesis makes it possible to assemble even with potentially high levels of variation [3]. Indeed, when SNP discovery is a major goal it is typically preferred to use an outbred individual. Seven Hawaiian honeycreeper samples were selected for RAD tag sequencing: one iiwi (*Vestiaria coccinea*; female RCF 2682, sampled 8 March 1987 at Kokee State Park, Kauai), two palila (*Loxioides bailleui*; bands 8031-75515 and 8031-75622, sampled in 1993 at Puu Laau, Hawaii Island), one apapane (*Himatione sanguinea*; 1540-45550 sampled at Waikimoi Preserve, Maui), one Hawaii amakihi (the same individual used for genome assembly), and two Nihoa finches (*Telespiza ultima*; bands 1381-62204 and 1381-62194 sampled on Nihoa Island, HI). This selection of honeycreepers covers much of the Drepanidine tree, and includes two redbird species (iiwi, apapane), two finchbill species (Nihoa finch, palila) and a greenbird (amakihi). Samples used in this study were obtained under appropriate USFWS and Hawaii DLNR-DOFAW permits, and IACUC approvals. For a recent phylogeny of Hawaiian honeycreepers, see Lerner et al. *Current Biology* 2011, 21:1838-1844.

### DNA isolation

Genomic DNA was extracted from whole blood using proteinase K digestion followed by phenol:chloroform extraction and either ethanol precipitation (Nihoa finches one palila) or Amicon® Ultra-4 (Millipore, Billerica, MA) centrifugal dialysis [51] (amakihi). Alternately, for iiwi, apapane, and the other palila, DNA was extracted using a Qiagen DNeasy Blood and Tissue kit (Qiagen, Germantown, MD). DNA quality and concentration were visualized using agarose gel electrophoresis and quantified using a NanoDrop 1000 spectrophotometer (NanoDrop, Wilmington, DE).

### 454 Library construction and sequencing

For 454 sequencing, ~10 ug of genomic DNA was fragmented using a HydroShear apparatus from Genomic Solutions Ltd, and 454 library preparation was done following manufacturer recommended protocols using the Titanium Rapid Library Preparation Kit, with insert sizes greater than 1000 bp. The libraries were then processed for shotgun Roche FLX+ sequencing in 4 lanes, to a total of 2.5X coverage. Average read length was 458 bp.

### Illumina library construction and sequencing

A total of 5 ug of input DNA was sheared by sonication (Covaris) and size-selected using a Pippin Prep (Sage Science). The fragmented DNA was end-repaired and ligated to Illumina adapters using a SPRI-TE robot and reagents (Beckman Coulter, Inc.). Illumina indexes were then added using 10-cycle PCR reaction performed in duplicate. The amplified library products were pooled and subjected to two rounds of Agencourt AMPure XP (Beckman Coulter, Inc.) bead clean up. The library was run on an Illumina MiSeq (v1 reagents) and two lanes of an Illumina HiSeq2000 (v3 reagents). The insert size of the library was subsequently determined by paired-end read mapping back to the genome assembly to be 392 +/- 29 bp.

### RAD tag library construction and sequencing

For the samples involved in RAD tag development, DNA samples were prepared for RAD tag sequencing generally following the protocol of Baird et al. (2008) [31], with modifications. These included the use of directional TruSeq-style adapters with 10 bp unique indices, and selecting a restriction enzyme with indeterminate bases at the cut site to accommodate requirements of Illumina HiSeq chemistry [52]. Briefly, 2 ug of genomic DNA for each sample was digested with the *Bse*XI enzyme, ligated to an adapter with a unique 10 bp index sequence, and sheared to approximately 300 – 500 bp fragments. A second adapter also containing the index sequence was ligated to the other end of the sheared fragments. Adapters were designed so that only fragments with adapters ligated to both ends would amplify. Each library was amplified using Phusion master mix (New England Biolabs, Ipswich, MA) for 15 – 18 cycles of PCR. Magnetic beads (Sera-Mag Speed Beads, Thermo Fisher Scientific, Waltham, MA) were used to purify libraries after amplification and filter out small fragments. Libraries were assessed for correct size and concentration using an Agilent BioAnalyzer. Samples were pooled in equimolar ratios and sequenced on an Illumina HiSeq with 100 bp paired-end reads (amakihi, iiwi, apapane and one palila) or MiSeq with 150 bp paired-end reads (both Nihoa finches and one palila). Paired-end sequencing generates two reads for each fragment, each starting from opposite ends of the fragment.

### Genome assembly and comparative analysis

Quality filtered Illumina reads (>80% of bases in the read pair had quality scores > 20) corresponding to ~19-fold coverage (assuming a 1 Gb genome) and filtered 454 reads (reads with at least 300 bp of Q20 bases) corresponding to ~2-fold coverage were used for a genome assembly with phusion [53]. Chromosome level scaffolds were generated from the assembled contigs by merging position and orientation information about a subset of the reads in the amakihi contigs with their orthologous position in the zebra finch genome (taeGut1) [2] as determined by a megablast [54] search. The amakihi chromosome level scaffolds were aligned to the zebra finch genome with Pecan [26] using the default settings. The consensus sequences for each chromosome have been uploaded to NCBI (BioProject 252695) and will be available upon publication of this article.

### SNP discovery in the amakihi genome

The Illumina reads were mapped to the amakihi genome assembly with Novoalign V2.08.02 (Novoalign short read mapper: http://www.novocraft.com/), duplicate read-pairs were removed using SAMtools [55] and variants detected using MPG [25]. For genome-wide statistics, single-nucleotide variants were filtered to include only heterozygous sites with an MPG score > =10 and a MPG score to read-depth ratio > = 0.5, and sites that had a read-depth less than approximately 2-fold the mean depth of coverage, i.e. <=100x on the autosomes and < =50x on the Z chromosome.

### Sequence processing using RAD tags without a reference

Raw reads were evaluated for quality using FastQC [56]. Reads were trimmed at the point where per-base quality score inter-quartile range dropped below a quality score of 20. The quality of most read two sequences deteriorated near the beginning of the read, so these sequences were not used. All read one sequences were trimmed to a length of 75 bp, the shortest length of any of the libraries before quality score dropped below 20. All reads were trimmed to this length because the Stacks RAD tag analysis software requires reads from all samples to be the same length. After they were trimmed, reads were filtered for quality using a python script (QualityFilterFastQ.py [57]) (amakihi, iiwi, apapane, both palila) or fastq_quality_filter from the FastX-toolkit [58] (both Nihoa finches), both of which removed any read that had any base pair with a quality score below 20.

Stacks [59] was used to assemble and call SNPs from RAD loci using the denovo_map.pl pipeline for samples without a reference genome. Several samples were first run individually using the populations mode of Stacks. Next, all samples were analyzed together using superparent mode. This mode is designed for test crosses and creates a catalog of possible loci based on the loci present in the parents. For non-cross samples, read one sequences are concatenated into a ‘superparent’ from which a catalog of stacks loci is developed, followed by alignment and genotyping of each sample at each catalog locus. Default parameters were used except as follows: minimum of three identical raw reads to create a stack and three mismatches allowed between loci when building the catalog of possible loci. The apapane read one file became corrupted during the compression process and was not used in analyses subsequent to individual Stacks runs. After running Stacks, Python scripts were used to filter the output to remove stacks that were found in the superparent catalog but not found in any progeny (samples; no progeny filter) or where one or more individuals had more than two genotypes for a given locus (bad genotypes filter). Stacks representing repetitive regions of the genome were removed by assembling the stacks consensus sequences with minimum overlap 70 bp and maximum read difference of 5% and then discarding stacks that assembled into contigs composed of greater than two sequences.

Using the quality-filtered Stacks consensus sequences only, we compared Stacks SNP calls for the amakihi with genotypes from the genome assembly (same amakihi). BWA was used to align Stacks consensus sequences to the genome assembly. Next, custom Python and Perl scripts were used to match Stacks SNP genotypes with genome genotypes on a sample of 11 chromosomes selected to include various sizes (chromosomes 1, 5, 7, 9, 15, 20, 22, 23, 24, 26 and 28). These scripts are available upon request to the author.

### Alignment of RAD reads to amakihi genome and SNP genotyping

Read one sequences from the RAD tag libraries were trimmed and quality filtered as for Stacks analysis, except reads from the MiSeq run (both Nihoa finch and one palila) were trimmed to 130 bp instead of 75 bp as there was no need to keep all sequences the same length for this part of the analysis. The amakihi genome assembly was indexed using the ‘bwtsw’ algorithm of BWA [37] and the trimmed, quality-filtered read one sequences were aligned to the indexed reference using the ‘samse’ algorithm [37] for single reads. The HaplotypeCaller function [60] of the Genome Analysis Toolkit (GATK [38]) was used to identify variable sites between the amakihi genome and aligned honeycreeper reads using the MalformedReadFilter and default parameters. The VariantFiltration function of GATK was used to filter variant sites, passing those with quality >30 and depth >6.

### Interspecies comparisons

All RAD read one sequences were aligned to the amakihi reference sequence using Geneious and calls for each sample for all sites were generated using the GATK HaplotypeCaller function with the EMIT_ALL_CONFIDENT_SITES parameter. PyRAD v. 1.2 [39] was used to identify RAD sequences with 10X or higher coverage present in three or more (out of seven) taxa. These were clustered based on similarity of 0.9 in USEARCH [61]. The total number of aligned base-pairs was 12,847. A maximum likelihood analysis in Garli v2.0 [40] was performed on these data with 100 search replicates.

### Availability of supporting data

The data sets supporting the results of this article, including the amakihi genome sequence (each chromosome sequence in FASTA format) and raw RAD reads (FASTQ format), are available in the NCBI repository, BioProject: PRJNA252695.

## Electronic supplementary material

Additional File 1: **Table showing counts for raw and quality-filtered RAD sequencing reads for each honeycreeper sample.** (DOCX 38 KB)

## References

[1] **International Chicken Genome Sequencing Consortium. Sequence and comparative analysis of the chicken genome provide unique perspectives on vertebrate evolution** *Nature* 2004, **432:**695–716. 10.1038/nature03154 10.1038/nature03154 15592404

[2] Warren WC, Clayton DF, Ellegren H, Arnold AP, Hillier LW, Kunstner A, Searle S, White S, Vilella AJ, Fairley S, Heger A, Kong L, Ponting CP, Jarvis ED, Mello CV, Minx P, Lovell P, Velho TAF, Ferris M, Balakrishnan CN, Sinha S, Blatti C, London SE, Li Y, Lin Y-C, George J, Sweedler J, Southey B, Gunaratne P, Watson M (2010). The genome of a songbird. Nature.

[3] Dalloul RA, Long JA, Zimin AV, Aslam L, Beal K, Ann Blomberg L, Bouffard P, Burt DW, Crasta O, Crooijmans RPMA, Cooper K, Coulombe RA, De S, Delany ME, Dodgson JB, Dong JJ, Evans C, Frederickson KM, Flicek P, Florea L, Folkerts O, Groenen MAM, Harkins TT, Herrero J, Hoffmann S, Megens H-J, Jiang A, de Jong P, Kaiser P, Kim H (2010). Multi-Platform Next-Generation Sequencing of the Domestic Turkey (Meleagris gallopavo): Genome Assembly and Analysis. PLoS Biol.

[4] Rands CM, Darling A, Fujita M, Kong L, Webster MT, Clabaut C, Emes RD, Heger A, Meader S, Hawkins MB, Eisen MB, Teiling C, Affourtit J, Boese B, Grant PR, Grant BR, Eisen JA, Abzhanov A, Ponting CP (2013). Insights into the evolution of Darwin’s finches from comparative analysis of the Geospiza magnirostris genome sequence. BMC Genomics.

[5] Ganapathy G, Howard JT, Ward JM, Li J, Li B, Li Y, Xiong Y, Zhang Y, Zhou S, Schwartz DC, Schatz M, Aboukhalil R, Fedrigo O, Bukovnik L, Wang T, Wray G, Rasolonjatovo I, Winer R, Knight JR, Koren S, Warren WC, Zhang G, Phillippy AM, Jarvis ED (2014). High-coverage sequencing and annotated assemblies of the budgerigar genome. GigaScience.

[6] Ellegren H, Smeds L, Burri R, Olason PI, Backstrom N, Kawakami T, Kunstner A, Makinen H, Nadachowska-Brzyska K, Qvarnstrom A, Uebbing S, Wolf JBW (2012). The genomic landscape of species divergence in Ficedula flycatchers. Nature.

[7] Lerner H, Fleischer R (2010). Prospects for the Use of Next-Generation Sequencing Methods in Ornithology. Auk.

[8] Griffin DK, Robertson LBW, Tempest HG, Skinner BM (2007). The evolution of the avian genome as revealed by comparative molecular cytogenetics. Cytogenet Genome Res.

[9] Hackett SJ, Kimball RT, Reddy S, Bowie RCK, Braun EL, Braun MJ, Chojnowski JL, Cox WA, Han K-L, Harshman J, Huddleston CJ, Marks BD, Miglia KJ, Moore WS, Sheldon FH, Steadman DW, Witt CC, Yuri T (2008). A Phylogenomic Study of Birds Reveals Their Evolutionary History. Science.

[10] Kawakami T, Smeds L, Backström N, Husby A, Qvarnström A, Mugal CF, Olason P, Ellegren H (2014). A high-density linkage map enables a second-generation collared flycatcher genome assembly and reveals the patterns of avian recombination rate variation and chromosomal evolution. Mol Ecol.

[11] Barker FK, Cibois A, Schikler P, Feinstein J, Cracraft J (2004). Phylogeny and diversification of the largest avian radiation. Proc Natl Acad Sci U S A.

[12] Lerner H, Meyer M, Hofreiter M, Fleischer R (2011). Multilocus resolution of the phylogeny and timescale in the extant adaptive radiation of Hawaiian honeycreepers. Curr Biol.

[13] James H, Olson S (1991). Descriptions of thirty-two new species of birds from the Hawaiian Islands: Part II. Passeriformes. Ornithol Monogr.

[14] Fleischer RC, McIntosh CE, Tarr CL (1998). Evolution on a volcanic conveyor belt: using phylogeographic reconstructions and K–Ar-based ages of the Hawaiian Islands to estimate molecular evolutionary rates. Mol Ecol.

[15] Price JP, Clague DA (2002). How old is the Hawaiian biota? Geology and phylogeny suggest recent divergence. Proc R Soc Lond B Biol Sci.

[16] Banko WE, Banko PC (2009). Historic Decline and Extinction. Conserv Biol Hawaii For Birds.

[17] van Riper CI, van Riper SG, Goff ML, Laird M (1986). The epizootiology and ecological significance of malaria in Hawaiian land birds. Ecol Monogr.

[18] Atkinson CT, Woods KL, Dusek RJ, Sileo LS, Iko WM (1995). Wildlife disease and conservation in Hawaii: Pathogenicity of avian malaria (Plasmodium relictum) in experimentally infected Iiwi (Vestiaria coccinea). Parasitology.

[19] Atkinson CT, Samuel MD (2010). Avian malaria Plasmodium relictum in native Hawaiian forest birds: epizootiology and demographic impacts on apapane Himatione sanguinea. J Avian Biol.

[20] Van Riper CI, Scott J (2001). Limiting factors affecting Hawaiian native birds. Stud Avian Biol.

[21] Woodworth BL, Atkinson CT, LaPointe DA, Hart PJ, Spiegel CS, Tweed EJ, Henneman C, LeBrun J, Denette T, DeMots R, Kozar KL, Triglia D, Lease D, Gregor A, Smith T, Duffy D (2005). Host population persistence in the face of introduced vector-borne diseases: Hawaii amakihi and avian malaria. Proc Natl Acad Sci U S A.

[22] Atkinson C, Dusek R, Woods K, Iko W (2000). Pathogenicity of avian malaria in experimentally-infected Hawaii Amakihi. J Wildl Dis.

[23] Jarvi S, Atkinson C, Fleischer R (2001). Immunogenetics and resistance to avian malaria in Hawaiian honeycreepers (Drepanidinae). Stud Avian Biol.

[24] Foster JT, Woodworth BL, Eggert LE, Hart PJ, Palmer D, Duffy DC, Fleischer RC (2007). Genetic structure and evolved malaria resistance in Hawaiian honeycreepers. Mol Ecol.

[25] Teer JK, Bonnycastle LL, Chines PS, Hansen NF, Aoyama N, Swift AJ, Abaan HO, Albert TJ, Margulies EH, Green ED, Collins FS, Mullikin JC, Biesecker LG (2010). Systematic comparison of three genomic enrichment methods for massively parallel DNA sequencing. Genome Res.

[26] Paten B, Herrero J, Beal K, Fitzgerald S, Birney E (2008). Enredo and Pecan: genome-wide mammalian consistency-based multiple alignment with paralogs. Genome Res.

[27] Burt DW, Bruley C, Dunn IC, Jones CT, Ramage A, Law AS, Morrice DR, Paton IR, Smith J, Windsor D, Sazanov A, Fries R, Waddington D (1999). The dynamics of chromosome evolution in birds and mammals. Nature.

[28] Backström N, Karaiskou N, Leder EH, Gustafsson L, Primmer CR, Qvarnström A, Ellegren H (2008). A Gene-Based Genetic Linkage Map of the Collared Flycatcher (Ficedula albicollis) Reveals Extensive Synteny and Gene-Order Conservation During 100 Million Years of Avian Evolution. Genetics.

[29] Jetz W, Thomas GH, Joy JB, Hartmann K, Mooers AO (2012). The global diversity of birds in space and time. Nature.

[30] Aslam M, Bastiaansen J, Elferink M, Megens H-J, Crooijmans R, Blomberg L, Fleischer R, Van Tassell C, Sonstegard T, Schroeder S, Groenen M, Long J (2012). Whole genome SNP discovery and analysis of genetic diversity in Turkey (Meleagris gallopavo). BMC Genomics.

[31] Baird NA, Etter PD, Atwood TS, Currey MC, Shiver AL, Lewis ZA, Selker EU, Cresko WA, Johnson EA (2008). Rapid SNP Discovery and Genetic Mapping Using Sequenced RAD Markers. PLoS ONE.

[32] Davey JW, Cezard T, Fuentes-Utrilla P, Eland C, Gharbi K, Blaxter ML (2013). Special features of RAD Sequencing data: implications for genotyping. Mol Ecol.

[33] McQueen HA, Fantes J, Cross SH, Clark VH, Archibald AL, Bird AP (1996). CpG islands of chicken are concentrated on microchromosomes. Nat Genet.

[34] Smith J, Bruley CK, Paton IR, Dunn I, Jones CT, Windsor D, Morrice DR, Law AS, Masabanda J, Sazanov A, Waddington D, Fries R, Burt DW (2000). Differences in gene density on chicken macrochromosomes and microchromosomes. Anim Genet.

[35] Federico C, Cantarella C, Scavo C, Saccone S, Bed’Hom B, Bernardi G (2005). Avian genomes: different karyotypes but a similar distribution of the GC-richest chromosome regions at interphase. Chromosom Res.

[36] Nikolajewa S (2005). Common patterns in type II restriction enzyme binding sites. Nucleic Acids Res.

[37] Li H, Durbin R (2009). Fast and accurate short read alignment with Burrows-Wheeler transform. Bioinformatics.

[38] McKenna A, Banks E, Sivachenko A, Cibulskis K, Kernytsky A, Garimella K, Altshuler D, Gabriel S, Daly M, DePristo M (2010). The Genome Analysis Toolkit: a MapReduce framework for analyzing next-generation DNA sequencing data. Genome Res.

[39] Eaton DAR (2014). PyRAD: assembly of de novo RADseq loci for phylogenetic analyses. Bioinformatics.

[40] Zwickl D (2006). Genetic algorithm approaches for the phylogenetic analysis of large biological sequence datasets under the maximum likelihood criterion. PhD Dissertation.

[41] Amadon D (1950). The Hawaiian honeycreepers (Aves, Drepaniidae). Bull AMNH.

[42] Richards LP, Bock WJ (1973). Functional Anatomy and Adaptive Evolution of the Feeding Apparatus in the Hawaiian Honeycreeper Genus Loxops (Drepanididae). Ornithol Monogr.

[43] Raikow R (1977). The origin and evolution of the Hawaiian honeycreepers (Drepanididae). Living Bird.

[44] Johnson NK, Marten JA, Ralph CJ (1989). Genetic Evidence for the Origin and Relationships of Hawaiian Honeycreepers (Aves: Fringillidae). Condor.

[45] Tarr CL, Fleischer RC (1993). Mitochondrial-DNA Variation and Evolutionary Relationships in the Amakihi Complex. Auk.

[46] Reding D, Freed L, Cann R, Fleischer R (2010). Spatial and temporal patterns of genetic diversity in an endangered Hawaiian honeycreeper, the Hawaii Akepa (Loxops coccineus coccineus). Conserv Genet.

[47] Reding DM, Foster JT, James HF, Pratt HD, Fleischer RC (2009). Convergent evolution of “creepers” in the Hawaiian honeycreeper radiation. Biol Lett.

[48] Orr N, Back W, Gu J, Leegwater P, Govindarajan P, Conroy J, Ducro B, Van Arendonk JAM, MacHugh DE, Ennis S, Hill EW, Brama PAJ (2010). Genome-wide SNP association–based localization of a dwarfism gene in Friesian dwarf horses. Anim Genet.

[49] Jones FC, Grabherr MG, Chan YF, Russell P, Mauceli E, Johnson J, Swofford R, Pirun M, Zody MC, White S, Birney E, Searle S, Schmutz J, Grimwood J, Dickson MC, Myers RM, Miller CT, Summers BR, Knecht AK, Brady SD, Zhang H, Pollen AA, Howes T, Amemiya C, Lander ES, Di Palma F, Lindblad-Toh K, Kingsley DM (2012). The genomic basis of adaptive evolution in threespine sticklebacks. Nature.

[50] Tarr CL, Conant S, Fleischer RC (1998). Founder events and variation at microsatellite loci in an insular passerine bird, the Laysan finch (Telespiza cantans). Mol Ecol.

[51] Slikas B, Jones IB, Derrickson SR, Fleischer RC (2000). Phylogenetic relationships of Micronesian white-eyes based on mitochondrial sequence data. Auk.

[52] Faircloth BC, Glenn TC (2012). Not All Sequence Tags Are Created Equal: Designing and Validating Sequence Identification Tags Robust to Indels. PLoS ONE.

[53] Mullikin JC, Ning Z (2003). The Phusion Assembler. Genome Res.

[54] Zhang Z, Schwartz S, Wagner L, Miller W (2000). A greedy algorithm for aligning DNA sequences. J Comput Biol J Comput Mol Cell Biol.

[55] Li H, Handsaker B, Wysoker A, Fennell T, Ruan J, Homer N, Marth G, Abecasis G, Durbin R (2009). The Sequence Alignment/Map format and SAMtools. Bioinforma Oxf Engl.

[56] *Babraham Bioinformatics - FastQC A Quality Control tool for High Throughput Sequence Data*. [http://www.bioinformatics.babraham.ac.uk/projects/fastqc/]

[57] Kircher M, Shapiro B, Hofreiter M (2012). Analysis of High-Throughput Ancient DNA Sequencing Data. Anc DNA. Volume 840.

[58] *FASTX-Toolkit*. [http://hannonlab.cshl.edu/fastx_toolkit/index.html]

[59] Catchen JM, Amores A, Hohenlohe P, Cresko W, Postlethwait JH (2011). Stacks: Building and Genotyping Loci De Novo From Short-Read Sequences. G3 Genes Genomes Genet.

[60] DePristo M, Banks E, Poplin R, Garimella K, Maguire J, Hartl C, Philippakis A, del Angel G, Rivas M, Hanna M, McKenna A, Fennell T, Kernytsky A, Sivachenko A, Cibulskis K, Gabriel S, Altshuler D, Daly MJ (2011). A framework for variation discovery and genotyping using next-generation DNA sequencing data. Nat Rev Genet.

[61] Edgar RC (2010). Search and clustering orders of magnitude faster than BLAST. Bioinformatics.

