# Supplementary material for: Genomic resources for the endangered Hawaiian honeycreepers
Source: BMC Genomics. 2014 Dec 12;15(1):1098. doi: 10.1186/1471-2164-15-1098 (PMC4300047; doi:10.1186/1471-2164-15-1098)
Supplement: Supplementary file 1 — Additional File 1: Table showing counts for raw and quality-filtered RAD sequencing reads for each honeycreeper sample. (DOCX 38 KB) [file 12864_2014_6902_MOESM1_ESM.docx]

**Summary of raw RAD data.**

| **Sample** | **Raw Read 1 Sequences** | **Quality-filtered Read 1 Sequences** |
| --- | --- | --- |
| Apapane | 16,017,647 | 7,331,429 |
| Iiwi | 43,630,304 | 16,380,184 |
| Amakihi | 50,069,524 | 24,263,032 |
| Palila_Tag1 | 6,869,311 | 3,023,108 |
| Palila_2 | 652,854 | 319,559 |
| Nihoa_Finch_1 | 1,098,237 | 772,863 |
| Nihoa_Finch_2 | 1,503,204 | 1,084,183 |
